# Effects of a Twelve-Week Weight Reduction Exercise Programme on Selected Spatiotemporal Gait Parameters of Obese Individuals

**DOI:** 10.1155/2017/4193256

**Published:** 2017-01-11

**Authors:** Joseph A. Jegede, Babatunde O. A. Adegoke, Oladapo M. Olagbegi

**Affiliations:** ^1^Department of Physiotherapy, Federal Medical Centre, PMB 1053, Owo, Ondo State, Nigeria; ^2^Department of Physiotherapy, College of Medicine, University of Ibadan, Ibadan, Nigeria

## Abstract

*Objectives*. This study was carried out to investigate the effects of twelve-week weight reduction exercises on selected spatiotemporal gait parameters of obese individuals and compare with their normal weight counterparts.* Methods*. Sixty participants (30 obese and 30 of normal weight) started but only 58 participants (obese = 30, normal weight = 28) completed the quasi-experimental study. Only obese group had 12 weeks of weight reduction exercise training but both groups had their walking speed (WS), cadence (CD), step length (SL), step width (SW), and stride length (SDL) measured at baseline and at the end of weeks 4, 8, and 12 of the study. Data were analysed using appropriate descriptive and inferential statistics.* Results*. There was significantly lower WS, SL, and SDL but higher CD and SW in obese group than the normal weight group at baseline and week 12. However, the obese group had significantly higher percentage changes in all selected spatiotemporal parameters than the normal weight group.* Conclusion*. The 12-week weight reduction exercise programme produced significantly higher percentage changes in all selected spatiotemporal gait parameters in the obese than normal weight individuals and is recommended for improvement of these parameters among the obese individuals with gait related problems.

## 1. Introduction

Obesity has been identified as a public health challenge [1] which is estimated as the fifth leading risk factor for several diseases such as increased incidence of cardiovascular disease, coronary heart diseases, pulmonary afflictions, type 2 diabetes mellitus (DM), hypertension, stroke, dyslipidemia, osteoarthritis, and cancers [2–5]. This condition which was once viewed as an affliction of the Western society has increased worldwide by more than 75% in the last three decades [6, 7]. Specifically, spatiotemporal gait alterations have been identified as one of the various negative consequences of obesity [8–14] which developed as adaptations to excess weight loading on the lower limbs while walking and later result in musculoskeletal injuries [4, 11, 12]. Body Mass Index (BMI) which is the weight in kilograms divided by the square of the height in meters (kg/m^2^) is considered as an appropriate measure for screening for obesity and its health risks [5]. BMI is used to classify individuals into normal weight (18.5–24.9 kg/m^2^), overweight (25–29.9 kg/m^2^), and obese (>30 kg/m^2^) categories (WHO, 2000). Obesity is further classified as class I (30–34.9 kg/m^2^), class II (35–39.9 kg/m^2^), and class III (>40 kg/m^2^) obesity [5].

The negative effects of obesity on gait have been widely reported in literature [15–18]. Studies that compared the gait characteristics of obese and normal weight individuals have reported that the obese individuals take shorter steps (1.25 m versus 1.67 m) and wider strides (0.16 m versus 0.08 m) than their normal weight counterparts [8, 19]. Other studies have also found that obese individuals have significantly lower preferred walking speed, reduced cadence, shorter step length, and wider step width than their normal weight counterparts [8, 16, 18, 20]. Ko et al. [10] evaluated the effects of obesity on some gait parameters using data from 164 older obese and nonobese adults and observed that spatiotemporal gait parameters were more affected by excess weight and therefore recommended that additional studies are needed to ascertain if losing weight will normalize gait pattern characteristics of obese individuals. However, studies on the effects of weight reduction on gait parameters are rather scarce despite the documented differences between gait parameters of obese and normal weight individuals as well as role of exercises in weight loss.

Although systematic reviews and other research reports [8, 18, 20, 21] have provided strong evidences in support of weight reduction programme as routine method used in the management of obesity, it has not been well documented if such interventions lead to any improvement in spatiotemporal gait parameters. If obesity results in altered gait parameters, it is reasonable to expect that weight loss should restore and improve gait parameters. This study was therefore designed to investigate the effects of a 12-week weight reduction exercise programme on selected spatiotemporal gait parameters (walking speed, stride length, cadence, step length, and stride width) of obese individuals. It was hypothesized that the spatiotemporal gait parameters of obese and normal weight individuals will differ significantly and that the exercise programme will significantly improve the obese individuals' spatiotemporal gait parameters.

### 1.1. Method

Purposive sampling technique was used to recruit participants into the obese (BMI ≥ 30 kg/m^2^) and normal weight (BMI 18–24.9 kg/m^2^) groups, respectively. Ethical approval of the Health Research Ethics Committee of the University of Ibadan/University College Hospital, Ibadan (ref no. NHREC/05/01/2008a) was sought and obtained before the commencement of the study. The study's procedure was explained to the participants and their informed consent was sought and obtained. The potential participants were initially screened using Physical Activity Readiness Questionnaire [22] and only those who satisfied the following conditions were considered eligible for this study:

1. obese individuals (BMI ≥ 30 kg/m^2^),
2. normal weight individuals (BMI 18–24.9 kg/m^2^),
3. individuals without musculoskeletal disorders like low back pain, osteoarthritis, and ankle injuries.

A minimum sample size of 52 (26 per group) was estimated for the study at *α* = 0.05, power = 80%, and effect size = 0.8 using Cohen's table [23]. Thirty obese and thirty normal weight people were however recruited into the study though only 58 (obese = 30 and normal weight = 28) completed the study giving an attrition rate of 3.4%.

### 1.2. Tests and Measurements

Participants' weight and height were assessed using standardized procedures and their BMI was then estimated using the following formula:

(1) $$\begin{array}{c} BMI=\frac{Weight\;\left(kg\right)}{{Height}^{2}\;\left(m^{2}\right)}. \end{array}$$

Participants were subsequently classified as obese and normal weight and assigned to the appropriate study groups. The resting heart rates of the obese participants were assessed using standardized procedures and their Target Heart Rate was determined using the following formula by Karvonen and Vuorimaa [24]:

(2) Target  HR=%  IntensityMaxHR  used×HRreserve+Resting  HR,Maximum  Heart  RateMaxHR=220−Participant's  Age  in  years,HRreserve=MaxHR−Resting  HR.

### 1.3. Measurement of Spatiotemporal Gait Parameters

Spatiotemporal gait parameters were obtained by the footprint method whereby the soles of the feet were smeared with delible ink and the participants walked at their comfortable walking speeds along a 10-meter walkway covered with white paper. This method is an adaptation of the protocol by Jingguang et al., as cited by Li et al. [25]. The starting point of walking was selected to allow at least three steps before reaching the white paper platform to ensure steady-state gait [18]. Trials were considered satisfactory when both feet were in full contact with each of the white paper platforms. Participants' walking speed was measured by having the participants walk a distance of 10 meters and divided by the time taken in seconds to cover the distance using stopwatch. It is recorded in meter per seconds. Participants' step length, stride length, and step width were subsequently obtained and measured from the footprints using a plastic long ruler which was calibrated from 0 to 75 cm. The cadence was calculated dividing the number of steps taken by participants to cover the 10-meter distance by the time taken and recorded in steps per minutes. The spatiotemporal gait parameters of all participants were assessed at baseline and at the end of week 4, week 8, and week 12 of the study [20].

### 1.4. Intervention

#### 1.4.1. Obese Group

Each participant was instructed to continue on their normal balanced diet and not to take any junk meal in between their daily meals. The researcher demonstrated all the activities to be performed before the commencement of the training sessions. The research assistants helped in monitoring and supervising activities carried out by participants to ensure that they were properly carried out. Obese participants performed the weight reduction exercise sessions on three alternate days each week after all the required baseline measurements have been taken. The exercise programme focused on aerobic exercises, stationary cycling on ergometer, and walking on the treadmill. The warm-up comprised flexibility and stretching exercises to the arms, legs, and trunk while cooling down comprised strolling around the research area. All the exercises were carried out at a safe level of moderate intensity between 60% and 70% of age predicted maximum heart rate. The obese participants' spatiotemporal gait parameters were assessed at baseline and at the end of weeks 4, 8, and 12 of the study. The exercise protocol [26] was in 3 phases which comprised the following.

*(A) Warm-Up Exercises*. The warm-up exercises which lasted for 5 minutes included the following: flexibility exercises, head rotations to the right and left, neck flexion and extension, shoulder shrugs/arms stretching, alternate leg bends, alternate leg stretch, trunk side bends, waist circles, strolling, and light jogging [27].

*(B) Aerobic Exercises (Main Menu)*

*Exercise 1*

*Type*. Sit-up exercise.

*Starting Position*. Supine lying on the sit-up bench with knees bent and hands clasped behind the neck.

*Instruction*. Participant was instructed to lift up the head and trunk from the lying to upright position, hold the position for about 10 seconds, and slowly lower the body to the initial lying position. The exercise was carried out for duration of 5 minutes (between baseline and week 4), 7 minutes (between week 4 and week 8), and 10 minutes (week 8 and week 12) with a rest period of 3 minutes before the next exercise.

*Exercise 2*

*Type*. Cycling.

*Starting Position*. Sitting on the bicycle ergometer with feet well placed on the pedals.

*Instruction*. Participants rode on the bicycle ergometer against zero resistance for about 5-minute, 8-minute, and 10-minute duration in baseline–week 4, week 4–week 8, and week 8–week 12, respectively, with a rest period of 3 minutes.

*Exercise 3*

*Type*. Treadmill exercises.

*Position*. Standing on the treadmill.

*Instruction*. Participant walked at a comfortable pace on the treadmill zero inclination and resistance for a period of 5 minutes (baseline–week 4), 10 minutes (week 4–week 8), and 18 minutes (week 8–week 12) with a rest period of 2 minutes.

*(C) Cooling Down*. The participant was asked to take a stroll around the research area for 5 minutes to achieve cool down.

*Exercise Intensity*. Prescribed intensity was 60–70% of the maximum heart rate (MHR). Each obese participant commenced the exercise training programme at 60% of heart rate reserve. Progression was made after every four weeks, ensuring 5% increment in the obese participants' exercise heart rate. This exercise intensity progressed continually until the upper limit of 70% of heart rate reserve was reached.

*Exercise Frequency*. Three (3) sessions a week (alternate days) were ensured throughout 12 weeks of the study.

#### 1.4.2. Normal Weight Group

The participants were not allowed to partake in any form of weight reduction exercise but had their walking speed, cadence, step length, step width, and stride length assessed at baseline and at the end of weeks 4, 8, and 12 of the study [20].

### 1.5. Data Analyses

The data were analyzed using SPSS 16.0 version software (SPSS Inc., Chicago, Illinois, USA). Descriptive statistics of mean, standard deviation, percentages, and tables were used to summarize the data. Independent *t*-test was used to compare the spatiotemporal gait parameters of obese and normal weight groups at baseline and end of weeks 4, 8, and 12 of the study. One-way ANCOVA was also used to compare the groups' parameters using baseline values as covariates. Repeated measures ANOVA was used for within-group comparison of obese group participants' walking speed, cadence, step length, step width, and stride length across baseline, week 4, week 8, and week 12 of the study while paired *t*-test with Bonferroni adjustment was used for post hoc analysis.

Level of significance was set at 0.05 and 0.0125 for between-group and within-group comparisons, respectively.

## 2. Results

The groups were not significantly different in their ages (*p* < 0.125) but the obese group was significantly shorter (*p* < 0.003) and weighed significantly more (*p* < 0.001) than the normal weight group (Table 1). The groups' variables at the different time points of the study are compared and presented in Table 2. The normal weight group had significantly lower values of walking speed, step length, and stride length at baseline but obese group had significantly higher values of cadence at all-time points except at week 12 and significantly wider step width at baseline and week 4 of the study. Since the groups were significantly different in their height, weight, and baseline parameters, one-way ANCOVA was done to compare the two groups using the baseline values as covariates; the results indicated that the groups were significantly different at twelfth week of the study (Table 3). Percentage changes in the groups' variables were compared and presented in Table 4; the obese group had significantly higher percentage changes in all selected parameters than their normal weight counterparts. Within-group comparison showed significant improvement in mean walking speed, cadence, step length, stride length, and step width in the obese group across the four time points of the study with the values becoming higher as the study progressed (Table 5).

## 3. Discussion

### 3.1. Comparison of Spatiotemporal Gait Parameters of Obese and Normal Weight Participants

At baseline, the obese participants demonstrated lower walking speed, shorter step length and stride length, wider step width, and higher cadence than their normal weight counterparts. The results were not unexpected because the obese participants had significantly higher weight than their normal weight controls according to BMI classification, and studies have indicated that increase in body weight is strongly linked with changes in majority of the components of normal gait due to a modification of the body geometry by the addition of mass to different regions which negatively influences the biomechanics of walking [9, 10, 14, 28]. Obesity has also been reported to be associated with altered spatiotemporal gait parameters such as lower gait speed, shorter strides, and increased step width, and a significantly higher metabolic cost of walking compared to people with normal body weight [10, 12, 14, 19, 28]. The findings of this study were hence in line with previous research findings.

Spyropoulos et al. [19] reported a slower walking speed and shorter stride and step lengths for all classes of obese adults compared to their normal weight counterparts. De Souza et al. [12] examined the gait parameters of class II and III obese individuals and compared them with that of their normal weight counterparts and observed that the obese participants exhibited a slower walking speed, reduced cadence, and reduced stride length. The mean baseline walking speed, step length, and stride length observed among obese participants in this study were in agreement with the findings of most previous studies except the cadence. The result of this study showed a significantly higher cadence in obese individuals compared to their normal weight counterparts which is at variance with other previous studies.

At the fourth week of the study, there were significant differences in walking speed, step length, step width, and stride length of the obese and normal weight groups but there was no significant difference between their cadences. The result implied that there were some improvements in the spatiotemporal gait parameters which could be attributed to the effects of the weight reduction exercise intervention. Despite these results, the walking speed, step length, and stride length of the obese participants were still found to be lower than those of their normal weight counterparts. However, the normal weight participants still had significantly lower cadence and step width than their obese counterparts.

The obese group had significantly higher cadence than the normal weight group but the two groups were comparable in their walking speed, step length, step width, and stride length at the eighth week of the study. This finding indicated that the weight reduction intervention undertaken by the obese participants has produced improvements in the aforementioned spatiotemporal gait parameters which made them comparable to those of their normal weight counterparts. There was paucity of studies on the effects of weight reduction programme on spatiotemporal gait parameters of obese individuals after a period of intervention; hence findings from this study could not be compared with reports from previous studies.

At the twelfth week, the groups were not significantly different in their walking speed, cadence, step length, step width, and stride length. It can hence be deduced from the findings of this study that the twelve-week weight reduction exercise programme produced desirable and positive effects on all the five selected spatiotemporal gait parameters of the obese individuals to the extent that they became comparable to those of their normal weight counterparts who did not undergo any exercise training at the end of the study. The result of a recent randomized controlled trial by Song et al. [29] indicated that the obese group had more significant reduction in step width than the normal group while the two groups remained comparable in terms of other tested spatiotemporal gait parameters following a twelve-week weight reduction programme. The difference between the work of Song et al. [29] and the findings of this study may be attributable to the mode of exercise used for weight reduction; their older participants only had unsupervised self-paced walking without any time limit while the obese participants in this study had supervised progressive graded exercises such as treadmill walking, bicycle ergometry, sit-up exercise, and self-paced walking.

Studies conducted by Saibene and Minetti [30] and Foster et al. [31] indicated that excessive amount of adipose tissue in obese individuals made their walking less efficient; the obese people hence needed more effort to overcome friction between thighs, arms, and torso and to perform clearance maneuvers (legs and arms swinging wide to move around thighs and torso, resp.). The observed positive effects of the weight reduction exercise programme from the fourth week of this study may hence be assumed as evidence in support of the aforementioned viewpoints.

### 3.2. Effects of Weight Reduction Exercise Programme on Selected Gait Parameters of the Obese Group across the Four Time Points of the Study

There were significant differences in the walking speed, cadence, step length, and step width of the obese participants across the four time points of the study (week 0, week 4, week 8, and week 12 of the study) with the gait parameters improving progressively as the study progressed. This implies that the 12-week weight reduction exercise programme was sufficient and capable of significantly improving walking speed, cadence, step length, step width, and stride length of obese individuals at the end of the study. Studies [13, 19] have shown that high intensity physical activities such as weight reduction exercises result in decreased body fat which ultimately resulted in reduction in body weight. Studies on the effects of weight reduction exercise programme on spatiotemporal gait parameters of obese are rather scarce but the findings of this study regarding the effects of weight reduction programme on spatiotemporal gait parameters are in agreement with the work of Plewa et al. [32] who investigated the effects of a 3-month weight reduction programme on some selected kinematic gait parameters (walking speed, cadence, stride length, stride time, stance time, swing time, and double support time) among 52 obese women. They reported significant increases in walking speed, cadence, and stride length but significant reductions in cycle time, stance, and double support phases. Gait alterations in the obese are caused by the excess body weight induced by their larger thighs and shanks [10, 16]; hence the obese individuals modify their gait patterns by walking at their preferred speed as a way of implementing a balancing strategy [28]. For instance, obese individuals compensate for their reduced waking speed with faster cadence.

### 3.3. Clinical Implication of the Findings from This Study

The outcome of this study indicated that a twelve-week weight reduction exercise programme was effective in improving walking speed, cadence, step length, step width, and stride length of obese individuals. This exercise programme could hence be adopted by health professionals and other related professionals to improve the spatiotemporal gait parameters of individuals with obesity related problems.

## 4. Conclusion/Recommendation

Obese individuals have poorer spatiotemporal gait parameters than their normal weight individuals to values similar to those of their normal weight individuals. Therefore, weight reduction exercise programme is recommended as a means of improving obesity related spatiotemporal gait abnormality.

## Figures and Tables

**Table 1:** Physical characteristics of obese and normal weight participants.

Variable	Obese group (*n* = 30)	Normal weight group (*n* = 28)	*t*	*p* value
	Mean ± SD	Mean ± SD		
Age (years)	32.0 ± 8.26	29.32 ± 6.06	1.557	0.125
Weight (kg)	96.42 ± 15.02	69.29 ± 4.77	9.135	<0.001^*∗*^
Height (m)	1.66 ± 0.06	1.71 ± 0.07	3.145	0.003^*∗*^
BMI (kg/m^2^)	35.03 ± 4.91	23.55 ± 1.17	12.06	<0.001^*∗*^

**∗** indicates significant difference at alpha = 0.05.

**Table 2:** Independent *t*-test comparison of obese and normal weight groups' walking speed and cadence at baseline week 4, week 8, and week 12 of the study.

Parameters	Time frame	Obese group (OAG) (*n* = 30)	Normal weight (NWAG) (*n* = 28)	*p* value
		Mean ± SD	Mean ± SD	
WS (m/s)	Week 0	1.09 ± 0.17	1.29 ± 0.17	<0.001^*∗*^
	Week 4	1.21 ± 0.26	1.29 ± 0.17	0.163
	Week 8	1.28 ± 0.31	1.35 ± 0.19	0.229
	Week 12	1.35 ± 0.32	1.35 ± 0.19	0.989

CD (steps/sec)	Week 0	14.47 ± 0.97	12.82 ± 0.39	<0.001^*∗*^
	Week 4	13.93 ± 0.83	12.82 ± 0.39	<0.001^*∗*^
	Week 8	13.13 ± 0.86	12.82 ± 0.39	<0.001^*∗*^
	Week 12	12.77 ± 0.63	12.82 ± 0.39	0.084

StpL	Week 0	58.68 ± 7.42	66.42 ± 6.51	<0.001^*∗*^
	Week 4	62.67 ± 8.23	66.42 ± 6.51	0.061
	Week 8	65.34 ± 8.38	67.91 ± 6.53	0.200
	Week 12	66.83 ± 7.81	67.91 ± 6.53	0.573

StpW	Week 0	13.67 ± 4.15	9.79 ± 1.78	<0.001^*∗*^
	Week 4	11.79 ± 3.19	9.79 ± 1.78	0.005^*∗*^
	Week 8	10.27 ± 2.53	9.55 ± 1.80	0.218
	Week 12	8.81 ± 1.81	9.55 ± 1.80	0.125

StrdL	Week 0	117 ± 14.86	133 ± 13.02	<0.001^*∗*^
	Week 4	126 ± 16.67	133 ± 13.02	0.073
	Week 8	130 ± 16.93	136 ± 13.09	0.073
	Week 12	134 ± 16.68	136 ± 13.10	0.716

*∗* indicates significant between group difference at alpha = 0.05.

WS = walking speed; CD = cadence; SL = step length.

SW = step width; SLT = stride length.

SD = standard deviation.

**Table 3:** Analysis of covariance (ANCOVA) for comparison of the groups' variables using baseline values as covariates.

Variables	Mean square	*F*	*p* value	Partial Eta-square
WS	0.848	56.313	<0.001^*∗*^	0.506
CD	6.613	76.139	<0.001^*∗*^	0.581
SL	427.126	49.471	<0.001^*∗*^	0.474
SW	54.300	32.605	<0.001^*∗*^	0.372
SLT	2016.177	43.800	<0.001^*∗*^	0.443

*∗* indicates significant difference at alpha = 0.05.

WS = walking speed; CD = cadence; SL = step length.

SW = step width; SLT = stride length.

**Table 4:** Comparison of the groups' percentage changes at baseline/week 12 intervals of the study.

Variables	OBG (*n* = 30)	NWG (*n* = 28)	*t*	*p* value
	Mean ± SD	Mean ± SD		
	(%)	(%)		
WS	22.67 ± 13.19	4.33 ± 2.67	7.218	<0.001^*∗*^
CD	14.26 ± 7.73	2.27 ± 1.70	8.026	<0.001^*∗*^
SL	11.59 ± 3.51	0.00 ± 0.00	17.46	<0.001^*∗*^
SW	14.81 ± 8.44	2.24 ± 1.66	7.733	<0.001^*∗*^
SLT	32.54 ± 14.19	2.54 ± 4.01	10.788	<0.001^*∗*^

*∗* indicates significant between group difference at alpha = 0.05.

WS = walking speed; CD = cadence; SL = step length.

SW = step width; SLT = stride length.

SD = standard deviation.

**Table 5:** Repeated measures ANOVA and paired *t*-test post hoc multiple comparison of obese group participants across the 4 time points of the study.

Parameter	Baseline	Week 4	Week 8	Week 12	*F*	*p* value
	Mean ± SD	Mean ± SD	Mean ± SD	Mean ± SD		
WS (m/s)	1.09 ± 0.17^a^	1.21 ± 0.26^b^	1.28 ± 0.31^c^	1.35 ± 0.32^d^	44.84	<0.001^*∗*^
CD (stp/s)	14.47 ± 0.97^a^	13.93 ± 0.83^b^	13.13 ± 0.86^c^	12.77 ± 0.63^d^	138.99	<0.001^*∗*^
SL (cm)	58.69 ± 7.42^a^	62.67 ± 8.23^b^	65.34 ± 8.38^c^	66.83 ± 7.82^d^	87.19	<0.001^*∗*^
SW (cm)	13.67 ± 4.15^a^	11.79 ± 3.19^b^	10.27 ± 2.53^c^	8.81 ± 1.81^d^	54.26	<0.001^*∗*^
SLT (cm)	117.35 ± 14.86^a^	125.63 ± 16.6^b^	129.55 ± 16.95^bc^	134.33 ± 16.68^c^	42.83	<0.001^*∗*^

*∗* indicates significant difference at alpha = 0.05.

Superscripts (a, b, c, and d) represent post hoc analysis.

For a particular variable, mean values with different superscripts are significantly (*p* < 0.05) different. Mean values with same superscript are not significantly (*p* > 0.05) different. The pair of cell means that is significant has different superscripts.

WS = walking speed; CD = cadence; SL = step length.

SW = step width; SLT = stride length.

SD = standard deviation.
